# Supplementary material for: The Growth of Extended Melem Units on g-C3N4 by Hydrothermal Treatment and Its Effect on Photocatalytic Activity of g-C3N4 for Photodegradation of Tetracycline Hydrochloride under Visible Light Irradiation
Source: Nanomaterials (Basel). 2022 Aug 26;12(17):2945. doi: 10.3390/nano12172945 (PMC9457853; doi:10.3390/nano12172945)
Supplement: Supplementary file 1 [file nanomaterials-12-02945-s001.zip › nanomaterials-1877557-supplementary.pdf]

nanomaterials

Supplementary Materials

The Growth of Extended Melem Units on g-C_3N_4 by Hydrothermal Treatment and Its Effect on Photocatalytic Activity of g-C_3N_4 for Photodegradation of Tetracycline Hydrochloride under Visible Light Irradiation

Thi Van Anh Hoang, Phuong Anh Nguyen, Won Mook Choi and Eun Woo Shin *

School of Chemical Engineering, University of Ulsan, Daehakro 93, Nam-gu, Ulsan 44610, Korea
* Correspondence: ewshin@ulsan.ac.kr; Tel.: +82-52-259-2253

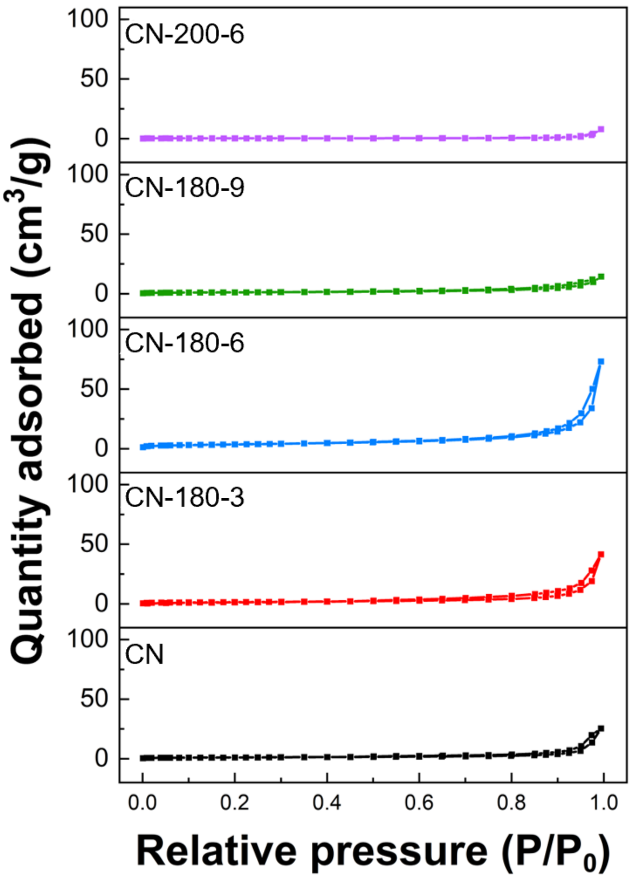

Figure S1. N_2 adsorption/desorption isotherms of as-prepared photocatalysts.

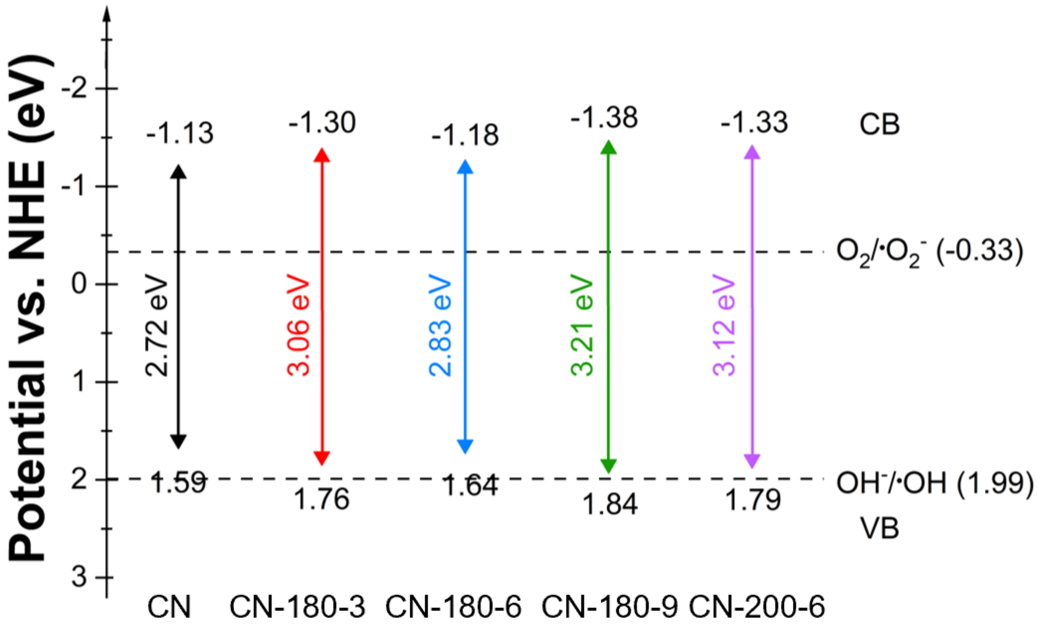

Figure S2. Band diagrams of the CN-180-x and CN-200-6 photocatalysts.

Table S1. O-groups data from XPS.

	CN-180-3	CN-180-6	CN-180-9	CN-200-6
**COOH**	25.10	47.30	51.86	46.24
**C=O**	24.02	40.95	39.48	24.56
**OH**	50.87	11.75	8.66	19.20

Table S2. Kinetic rate constants (k) and correlation coefficients (r^2) of TC photodegradation.

Sample	$k \times 10^3$ (min^{-1})	r^2
**CN**	6.6	0.98247
**CN-180-3**	11.8	0.99719
**CN-180-6**	23.6	0.98859
**CN-180-9**	5.8	0.99199
**CN-200-6**	2.3	0.99224
